# *Blautia coccoides*-derived metabolite trimethylamine-N-oxide exacerbates Alzheimer's disease progression via targeting HIF1α signaling

**DOI:** 10.1080/19490976.2025.2605768

**Published:** 2025-12-29

**Authors:** Xinhuang Lv, Tao Ye, Xiaolan Liao, Qiyao Li, Zheyu Fang, Xiaoou Lin, Mozi Chen, Conghui Dai, Lu Zhan, Linpei Zhuo, Kun Xiang, Jing Sun, Jiaming Liu

**Affiliations:** a Department of Neurology, The Second Affiliated Hospital of Wenzhou Medical University, Wenzhou, China; b Department of Preventive Medicine, School of Public Health, Wenzhou Medical University, Wenzhou, China

**Keywords:** Alzheimer's disease, *Blautia coccoides*, trimethylamine N-oxide, hypoxia-inducible factor 1 alpha, oxidative stress

## Abstract

An increasing number of studies have shown that commensal gut microbes may be involved in the pathogenesis of Alzheimer's disease (AD). The influence of gut microbe-derived metabolites, such as trimethylamine N-oxide (TMAO), has attracted a lot of attention. However, the influence and pathways mediated by gut microbe-derived metabolites in the pathogenesis of AD remain uncertain. Here, we observed a significant increase in the abundance of *Blautia coccoides* in AD patients, which showed positive predictive value for serum p-Tau181 levels. Supplementation with *B. coccoides* could exacerbate cognitive impairment and Tau phosphorylation in P301s mice. We identified TMAO as a key *B*. *coccoides*-derived metabolite promoting Tau phosphorylation by functional gene analysis, metabolomic analysis and VIP analysis, and further demonstrated that it was able to promote oxidative stress of AD *in vitro*. Mechanistically, TMAO could bind to hypoxia-inducible factor 1 alpha (HIF1α) at 235–238 sites, which promoted oxidative stress through the inhibition of HIF1α signal, thereby aggravating AD pathology. This study elucidated the important role of *B. coccoides*-derived metabolite TMAO in exacerbating AD and provided new insights for gut microbe/metabolite-based therapeutic strategies.

## Article highlights

*Blautia coccoides* abundance was increased in AD patients compared to healthy controls.

Supplementation with *B. coccoides* exacerbated AD pathology in model mice.

TMAO as a key *B. coccoides*-derived metabolite contributing to AD.

TMAO could directly bind to HIF1α, thereby inhibiting HIF1α signaling.

TMAO mediated *B. coccoides*-host crosstalk and was involved in AD pathology.

## Introduction

Alzheimer's disease (AD) is an incurable neurodegenerative disorder and the most prevalent form of dementia in elderly individuals, characterized by progressive cognitive impairment.^1^,^2^ The etiology of AD is very complex, involving amyloid β (Aβ) deposition, tau protein hyperphosphorylation, neuroinflammation and oxidative stress, and environmental factors.^3^,^4^ To date, the mechanism underlying neurodegeneration in AD remains unclear. Accumulating evidence suggests that the gut microbiota plays a crucial role in the pathogenesis of AD.^5^,^6^ Most of these studies relied on observational data without biological validation,^7^,^8^ thereby lacking causal inference. Gut microbiota alterations and their metabolites were associated with AD pathogenesis.^9^,^10^ Supporting this connection, fecal microbiota transplantation from AD transgenic mice to wild-type control mice resulted in AD-associated tau phosphorylation and cognitive impairment;^11^ conversely, transplantation of healthy microbiota could reduce AD pathogenesis.^12^ A recent study showed that alterations in the composition of the gut microbiota result in the peripheral accumulation of microbial metabolites, which are found to be associated with the increase in reactive oxygen species levels in neurogenerative disease.^13^ The contribution of gut commensal strains to AD pathogenesis remains largely unknown. Therefore, determining the relationship between specific strains and AD is critical for understanding the etiology and pathogenesis of AD.

Recently, an increasing evidence has demonstrated elevated abundance of *Blautia* genus in AD patients compared to healthy controls,^14^,^15^ which was associated with cognitive impairment.^16^ *Blautia* is a genus of anaerobic bacteria that is commonly found in gastrointestinal tracts including 20 species such as *Blautia coccoides*, *Bacteroides wexlerae, Bacteroides hansenii* and *Bacteroides producta*. Recent studies have indicated that the composition of and changes in the *Blautia* population in the gut are related to factors such as host age, diet, and disease status.^17^,^18^ *B. coccoides* is a representative species of *Blautia* genus,^19^ which is considered to have important pathogenic characteristics.^20^ It was reported that *B. coccoides* affects abnormal glucose and insulin homeostasis in patients with metabolic syndrome through its metabolites.^21^,^22^ However, whether *B. coccoides* contributes to AD progression or cognitive impairment remains largely unknown. Gut microbial-derived metabolites are a key focus in elucidating the mechanisms through which the specific microbes affect brain physiology and pathology. Specific microbes-derived metabolites, such as trimethylamine N-oxide (TMAO) and lipopolysaccharide (LPS), contribute to AD pathogenesis^13^ and serve as mediators in host-microbiota crosstalk.^23^,^24^ TMAO, a small organic molecule derived from by gut microbiota, can cross the blood‒brain barrier (BBB) to reach the brain,^25^ which was associated with an increased risk of AD.^26^ In our preliminary analysis, among several metabolites enriched in B. coccoides cultures, TMAO was identified as a key candidate owing to its known link to oxidative stress. However, so far, few microbial metabolites have been discovered and validated in the development of AD. Thus, more research is required to uncover role of microbe and its metabolites in the pathogenesis of AD.

In this study, we aimed to investigate the roles of *B. coccoides* derived metabolites in AD development. We evaluated the changes in *B. coccoides* abundance in AD patients and their correlation with AD pathology. We further elucidated the pathogenic effects of *B. coccoides* on cognitive impairment and Tau phosphorylation in AD model animals. Furthermore, we identified TMAO as the essential *B. coccoides*-derived metabolite contributing to AD, as well as elucidating the underlying pathogenic mechanisms. These findings present critical evidence for microbe‒host interactions and provide promising intervention targets for addressing AD.

## Materials and methods

### Subject recruitment and sample collection

**Figure 1.**
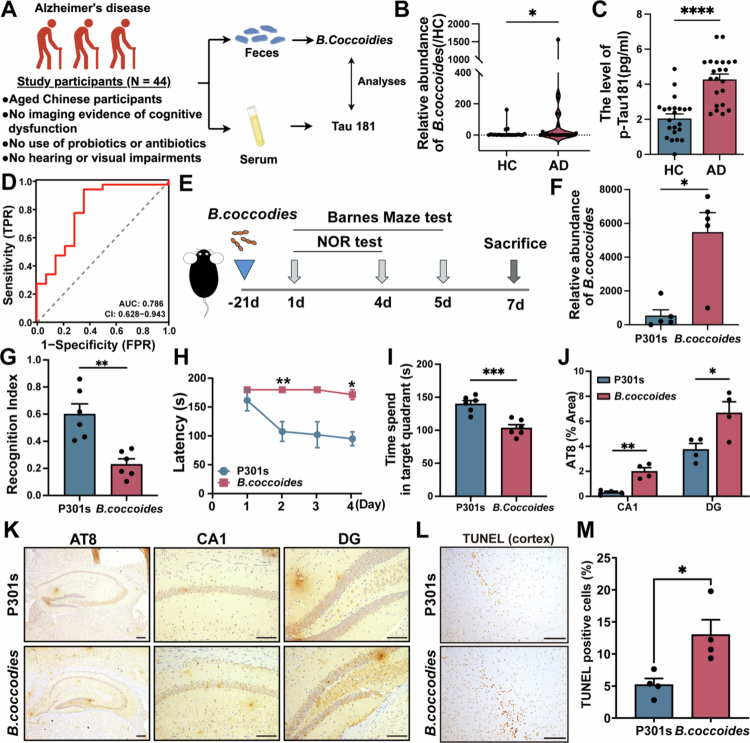
*B. coccoides* abundance was correlated with p-Tau level in AD patients, and *B. coccoides* supplementation aggravated cognitive function and pathological changes in P301s mice. (A) The experimental schematic diagram of cohort. (B) The comparison of *B. coccoides* abundance between AD patients and HC (*n* = 22). (C) The comparison of serum p-Tau181 level between AD patients and HC (*n* = 22). (D) Predictive capability of *B. coccoides* in serum p-Tau181 level of AD patients. (E) The experimental schematic diagram of animals. (F) The comparison of *B. coccoides* level between P301s and *B. coccoides* group (*n* = 6). (G) Comparisons of the discriminant index between P301s and *B. coccoides* group (*n* = 6). (H) Comparisons of latency in Barnes maze test between P301s and *B. coccoides* group (*n* = 6). (I) Comparisons of distance in target quadrant in Barnes maze test between two groups (*n* = 6). (J) Quantitative analysis of AT8 level between two groups (*n* = 4). (K) Representative immunohistochemical images of AT8 in P301s and *B. coccoides* group. The image above: magnification 50× and 200×. 50× Scale bar = 200 μm, and 200× Scale bar = 100 μm. (L) Representative immunohistochemistry images of TUNEL staining in two groups. The image above: magnification 200× and the scale bar = 100 μm. (M) Quantitative analysis of TUNEL^+^ cells in (H, *n* = 4). The error bars indicate the SEM, *p < 0.05, **p < 0.01, and ***p < 0.001.

This study included 22 AD patients and 22 age-matched healthy controls (HCs). AD was diagnosed according to the 2020 Chinese guidelines, with inclusion criteria of Minimum Mental State Examination (MMSE) <27 and Montreal Cognitive Assessment (MoCA) <26. HCs had no memory complaints, cognitive impairments, or major illnesses. None of the participants had taken antibiotics within two months or had serious metabolic disorders. Fasting blood and fecal samples were collected in the morning. Serum was obtained by centrifugation at 3000 rpm for 10 min at 4 °C, and fecal samples were snap-frozen in liquid nitrogen and stored at −80 °C. The study was approved by the Ethics Committee of the Second Affiliated Hospital of Wenzhou Medical University. Participant recruitment is shown in Figure 1A.

### Bacteria culture

*Blautia coccoides* (*B. coccoides*, ATCC, 29236) was cultured under anaerobic conditions at 37 °C using a modified chopped meat medium (ATCC Medium 1490) supplemented with vitamin K1 (1 mg/L, Beyotime, China) and hemin (5 mg/L, Shandong Tuopu Biol-engineering Co., Ltd, China).

### Animal and treatment

Male adult MAPT P301S transgenic mice (7-month-old) were purchased from Hangzhou Ziyuan Experimental Technology Co., Ltd., Hangzhou, China. The animals were accommodated in a temperature-controlled facility with a 12 h light/dark cycle and provided with standard chow and water without restriction. P301S transgenic mice were randomly divided into PBS-treated group and *B. coccoides* -treated group (*n* = 6/group). After confirming the activity, 1 × 10^9^ CFU/mL *B. coccoide* or PBS was daily gavaged to mice for 21 d. The experimental protocols were reviewed and endorsed by the Animal Experimentation Ethics Committee of Wenzhou Medical University.

### Detection of *B. coccoides*

The relative abundance of *B. coccoides* in the fecal samples was determined by quantitative PCR (qPCR). Genomic DNA was extracted using the alkaline lysis method, as described in our previous study. The bacterial abundance was calculated using the 2^–ΔΔCt^ and expressed relative to the HC or P301s group. The primer sequences are listed in Table S1.

### Object recognition test

The object recognition test was performed according to our previous study.^27^,^28^ The test was performed in a standard open-field box. 24-h prior to testing, each mouse was allowed to freely explore the box containing two identical rectangular wooden blocks for 5 min. During the test phase, one of a novel cylindrical block was replaced, and the time spent exploring the novel (TN) and familiar (TF) objects was recorded. The discrimination index (DI) was calculated as: DI = TN/(TN + TF) × 100%.

### Barnes maze test

The Barnes maze test was performed according to our previous study.^28^ The Barnes maze was conducted on a circular platform with 20 equally spaced escape holes, one of which led to a black escape box. The test spanned 6d and was divided into three phases: adaptation (day 1st), training (day 2nd–5th), and testing (day 6th). For adaptation, the mice were gently placed in the target hole and allowed to explore the escape box for 1 min. In the training phase, mice were briefly confined in a plastic cylinder (20 cm diameter, 27 cm height) at the maze center for 5 s. After removal of the cylinder, mice were given 3 min to locate the escape box, with the latency (time taken for all four limbs to enter) recorded. In the testing phase, the maze was divided into four quadrants, with the escape box positioned in the target quadrant's center. The escape box was then removed, and the time spent in the target quadrant was recorded.

### Pathology analysis

The mice were euthanized, and the brain samples were collected as described previously.^29^ The brain tissues were fixed in 4% paraformaldehyde at 4 °C for 48 h, embedded in paraffin, and sectioned at 5 μm. The sections were dried at 37 °C overnight and then baked at 65 °C for 150 min. After deparaffinization and blocking with 5% BSA, sections were incubated overnight at 4 °C with the following primary antibodies: AT8 (1:200, Thermo Fisher, MN1020), ZO-1 (1:200, Santa Cruz, sc-33725), and Occludin (1:2000, Proteintech, 27260-1-AP). Following secondary antibody incubation and DAPI staining, sections were observed under a light microscope (Leica, Germany), and brown granules indicated positive cells. TUNEL staining was performed with One Step TUNEL Apoptosis Assay Kit (Beyotime, China), following the established protocols. Fluorescence microscope and image analysis software were used to quantify the number of TUNEL-positive cells.

### Gut microbiota analysis

Fresh fecal sample was collected from the mouse for 16S rRNA sequencing, as previously described.^30^ Microbial DNA was extracted using the QIAamp DNA Stool Mini Kit (Qiagen, Germany). The V3–V4 region of the 16S rRNA gene was amplified using primers 338F and 806R, and the PCR products were purified and quantified. Sequencing was performed on the Illumina MiSeq platform by Majorbio Bio-Pharm Technology Co., Ltd. (Shanghai, China). High-quality reads were denoised and processed using QIIME2 (v2022.2) with the DADA2 plugin to generate amplicon sequence variants (ASVs). Taxonomic assignment was performed using a naïve Bayes classifier trained on the SILVA 138 database (https://www.arb-silva.de/) with a confidence threshold of 0.7. Alpha diversity was assessed by the Shannon and Simpson indices, and beta diversity was evaluated using principal coordinate analysis (PCoA).

### Two-sample Mendelian randomization analysis

Plasma TMAO-associated genetic data were obtained from the GWAS Catalog (ID: GCST90318598), comprising 1016 participants. Dementia in Alzheimer's disease data were sourced from the FinnGen consortium (finn-b-F5_ALZHDEMENT), including 2191 cases and 209,487 controls. Single nucleotide polymorphisms (SNPs) significantly associated with plasma TMAO levels (*p* < 1 × 10^–5^) were selected as instrumental variables (IVs). To ensure independence, linkage disequilibrium (LD) clumping was performed (*r*² < 0.001, window >10,000 kb), and SNPs with minor allele frequency (MAF) <1% were excluded to reduce pleiotropy and weak instrument bias. Instrument strength was evaluated using the *F*-statistic: *F* = (β_exposure/SE_exposure)², with SNPs retained only if *F* > 10. Mendelian randomization (MR) analyses were primarily conducted using the inverse variance weighted (IVW) method to assess the potential causal relationship between plasma TMAO levels and Dementia in Alzheimer's disease. Horizontal pleiotropy was evaluated using the MR-Egger intercept test, and heterogeneity among SNPs was assessed with Cochran's *Q* statistic. Sensitivity analysis was performed using the leave-one-out approach. All the statistical analyses were conducted in *R* (v4.4.1) using the TwoSampleMR package (v0.6.8).

### Cell culture and transfection

The Neuro-2a and HEK-293T cells were purchased from the Cell Resources Center of the Shanghai Institute of Biosciences (Chinese Academy of Sciences, Shanghai, China). The cells were cultured in DMEM (Corning, NY, USA) supplemented with 10% (v/v) FBS (Sigma, Carlsbad, CA, Australia) and maintained at 37 °C in a 5% CO_2_ incubator. P301L-tau441 (MAPT P301L, pcDNA3.1-His-C), HIF1α-WT (pcDNA3.1-3XFlag-C) and HIF1α-mut (pcDNA3.1-3XFlag-C) plasmids and respective control vectors were provided by RepoBio Biology (Hanzhou, China). For plasmid transfection, Neuro-2a or HEK-293T cells at 50% density were transfected with plasmid using PEI Transfection Reagent (Polysciences, USA) according to standard protocols. The medium was replaced 16–18 h post-transfection for further treatment.

### Cell viability assay

Cell viability was analyzed using a Cell Counting Kit-8 (CCK-8, GLPBIO, USA). In brief, cells (5 × 10^3^) were seeded into a 96-well plate and allowed to adhere and grow for a specified period. After 24 h of TMAO treatment, the medium was replaced with fresh medium containing 10% CCK-8 solution, and the cells were cultured for an additional 2 h. Cell viability was assessed by measuring the absorbance at 450 nm.

### ROS detection

Intracellular ROS production was determined using Reactive oxygen species Assay Kit (E004-1-1, Jiancheng, Nanjing, China). The cells were incubated with 2,7-dichlorofuorescin diacetate (DCFH-DA, 1:1000) at 37 °C for 10 min. The fluorescence intensity was measured using a FACS flow cytometer (BD Biosciences, USA).

### Hypoxia induction

The cells were pretreated with TMAO (MCE, USA) for 16 h. After pretreatment, the cells were exposed to 1% O₂ hypoxic conditions for an additional 6 h to stabilize HIF1α expression.

### LC‒MS

Fecal samples of mice were extracted with 400 μL of 80% methanol containing four internal standards. The mixture was homogenized using a Wonbio-96c high-throughput tissue grinder (Shanghai Wanbo Biotechnology Co., Ltd.) at 50 Hz and 10 °C for 6 min, followed by sonication for 10 min and centrifugation at 13,000 rpm for 15 min at 4 °C. The resulting supernatant was transferred to vials with inserts for LC‒MS analysis. Additionally, 20 μL of supernatant from each sample was pooled to generate a quality control (QC) sample. Metabolomic analysis was conducted using a Thermo Fisher UHPLC-Q Exactive HF-X system. Chromatographic separation was achieved on an ACQUITY UPLC HSS T3 column (100  × 2.1 mm, 1.8 μm; Waters, Milford, USA), with mobile phase A composed of 95% water and 5% acetonitrile (containing 0.1% formic acid) and mobile phase B composed of 47.5% acetonitrile, 47.5% isopropanol, and 5% water (also with 0.1% formic acid). The injection volume was 3 μL, and the column temperature was set at 40 °C. Electrospray ionization (ESI) was used for ionization, and data were acquired in both positive and negative ion modes.

### Western blot

Brain tissues of mice or cells samples were lysed in RIPA buffer (Beyotime, Shanghai) containing protease (ST506, Beyotime) and phosphatase inhibitors (P1260, Solarbio, Beijing), followed by centrifugation at 12,000 × *g* for 15 min at 4 °C. The supernatants were collected, and the protein concentration was determined using a BCA kit (Beyotime, Shanghai). Protein samples were mixed 1:1 with loading buffer (AR0131, BOSTER) and boiled at 95 °C for 5 min, and 20 μg of total protein was separated by SDS‒PAGE and then transferred to PVDF membranes. The membranes were blocked with 5% BSA at room temperature for 1 h and incubated overnight at 4 °C with the following primary antibodies: Occludin (1:1000, Bioworld, BS72035), HIF1α (1:1000, HuaBio, HA721997), HO-1 (1:1000, Bioworld, BS6626), SOD2 (1:1000, Bioworld, BS6734), GPX4 (1:1000, Bioworld, BS7323), p-Tau396 (1:1000, HuaBio, ET1611-68), p-Tau181 (1:1000, ABclonal, AP1324), Tau (1:500, Bioworld, BS1358), and Flag (1:2000, ABclonal, AE063). After washing, the membranes were incubated with HRP-conjugated secondary antibodies for 1 h at room temperature. The signals were developed and analyzed by grayscale intensity.

### ELISA assay

Blood samples from the subjects were collected and centrifuged to obtain the serum. Standards and serum samples were added to 96-well plates pre-coated with anti-Tau181 antibodies (Konodi Biotech, China) and incubated at 37 °C for 1 h. After washing with 0.05% Tween-20 in PBS, enzyme-conjugated secondary antibodies were added, and incubated for another hour at 37 °C. The plates were washed three times, followed by the addition of TMB substrate for color development at 37 °C for 15 min. The reaction was stopped with 2 M sulfuric acid, and the absorbance was measured at 450 nm using a microplate reader. Tau181 concentrations were calculated based on the standard curve and expressed in pg/mL.

### TMAO quantification

Quantitative analysis of TMAO was performed using targeted metabolomics. Standards were obtained from Shanghai Majorbio Bio-pharm Biotechnology Co., Ltd. (Shanghai, China), and each compound (1 mg) was dissolved in 1 mL of 50% acetonitrile-water to prepare stock solutions. Gradient working solutions were prepared by serial dilution, with TMAO calibration concentrations ranging from 6.25 to 5000 ng/mL. The internal standards were mixed at defined ratios and diluted to working concentrations in 50% acetonitrile-water. For sample preparation, 10 μL of internal standard and 10 μL of serum sample were added to 700 μL of extraction system in a 96-well plate, followed by brief centrifugation (10 °C, 4000 rpm, 10 s). The samples were then extracted with 180 μL of 90% acetonitrile, shaken at 800 rpm for 15 min at room temperature, and centrifuged again (10 °C, 4000 rpm, 15 min). The supernatant (100 μL) was transferred to LC‒MS vials for analysis. Chromatographic separation was carried out using a Waters BEH HILIC column (100 × 2.1 mm, 1.7 μm) on an ExionLC AD UHPLC system, and mass spectrometric detection was performed using a SCIEX QTRAP 6500+ (AB Sciex, USA) operating in positive ESI mode (CUR 35, IS + 5500 V, TEM 500 °C). The mobile phases consisted of 10 mM ammonium formate with 0.15% formic acid in water (A) and 95% acetonitrile (B), with a flow rate of 0.8 mL/min and an injection volume of 1 μL. Data were processed using Sciex OS software, and metabolite concentrations were determined based on peak area ratios to internal standards using linear regression from standard calibration curves. The results are expressed in ng/mL.

### Target prediction

The predicted targets of TMAO were retrieved from the Super-PRED database (https://prediction.charite.de/). To identify hub genes, protein–protein interaction (PPI) networks were constructed using the STRING database (https://string-db.org/) and further analyzed with the Cytoscape plug-in CytoHubba. DEGs were subjected to GO and KEGG pathway enrichment analyses using R software.

### RNA-seq and data analysis

Total RNA was extracted from TMAO-treated Neuro-2a cells using TRIzol® Reagent (Invitrogen) according to the manufacturer's instructions. The RNA quality was assessed, and the mRNA was isolated, fragmented, and reverse transcribed. cDNA library construction and Illumina sequencing were performed by Hangzhou Cosmos Wisdom Biotech Co., Ltd. Raw sequencing data were processed with fastp, and high-quality clean data were assembled using Trinity. The initial assembly was optimized with TransRate and validated using BUSCO. Differentially expressed genes (DEGs) were identified with edgeR, with genes having *p* < 0.05 and |log2FC| ≥ 1.2 considered significant. Functional enrichment was assessed using GO (Gene Ontology, http://www.geneontology.org/) and KEGG (Kyoto Encyclopedia of Genes and Genomes, http://www.genome.jp/kegg/) analyses, with FDR < 0.05 deemed significant.

### Molecular docking

The 3D structure of TMAO was downloaded from PubChem and minimized in ChemBio3D Ultra 14.0. The HIF1α crystal structure (AF-Q16665) was retrieved from AlphaFold and processed in PyMOL 2.3.0 to remove the original ligand. The structure was then analyzed using AutoDockTools, and protein binding sites were predicted with POCASA 1.1. Molecular docking between TMAO and HIF1α was performed using AutoDock Vina 1.1.2.

### Statistical analysis

All the data were statistically analyzed using SPSS V.26 (Chicago, USA) and visualized with GraphPad Prism V.10.1.1 (La Jolla, CA, USA). For normally distributed data, the results were expressed as mean ± SEM and were analyzed using one-way or two-way ANOVA followed by Tukey's or Dunnett's multiple comparison test or *t*-test two-group comparisons. Non-normal data were evaluated using the Mann‒Whitney test. Correlations were assessed by Spearman analysis. *p*-value < 0.05 was considered statistically significant.

## Results

### *B. coccoides* abundance was elevated in AD patients and *B. coccoides* supplementation aggravated pathogenesis in P301S mice

We determined that the fecal *B. coccoides* abundance and serum p-Tau181 level from AD patients and age-matched HC. Our findings revealed that the abundance of *B. coccoides* was significantly higher in AD patients compared to HC (*p* < 0.05, Figure 1B). Furthermore, we observed significantly higher serum p-Tau181 levels in AD patients than in HCs (*p* < 0.001, Figure 1C). To explore the predictive value of *B. coccoides* for AD, ROC curve analysis was performed based on the abundance of *B. coccoides*. Our results revealed that *B. coccoi*des showed predictive value for p-Tau181 level in AD patients, with an AUC of 0.786 (95% CI: 0.628–0.943, Figure 1D). These findings suggested that *B. coccoides* may be involved in AD progression.

To further investigate whether *B. coccoides* impacts AD progression, the P301S mice was fed with *B. coccoides* for 21 d. In the animal experiments, the qPCR results showed that *B. coccoides* could colonize in P301s mice (*p* < 0.05, Figure 1F). The intervention with *B. coccoides* yielded a notable aggravation of cognitive function in P301s mice. According to the results of the ORT, the discriminant index was significantly decreased in *B. coccoides*-treated mice compared with P301s group (*p* < 0.05, Figure 1G). The results of the Barnes maze test and the latency of *B. coccoides*-treated mice were significantly longer than P301s mice at the 3rd and 4th day (*p* < 0.01, Figure 1H), and the time spent in the target quadrant of *B. coccoides*-treated mice was significantly reduced (*p* < 0.01, Figure 1I). Taken together, the results indicated that *B. coccoides* treatment significantly impaired the spatial learning and memory of the P301s mice.

Cognitive impairment is often accompanied by the pathological changes of AD, which are characterized as tau protein hyperphosphorylation.^31^ According to the results of AT8 immunohistochemical staining, *B. coccoides* treatment significantly increased the level of Tau phosphorylation in P301s mice (*p* < 0.05, Figure 1J and K). The results of TUNEL staining, *B. coccoides* treatment significantly increased the number of Tunel^+^ cells in P301s mice (*p* < 0.01, Figure 1L and M). The findings indicated that *B. coccoides* could aggravate pathological damage in P301s mice.

### TMAO derived from *B. coccoides* as a potential factor in AD pathogenesis

**Figure 2.**
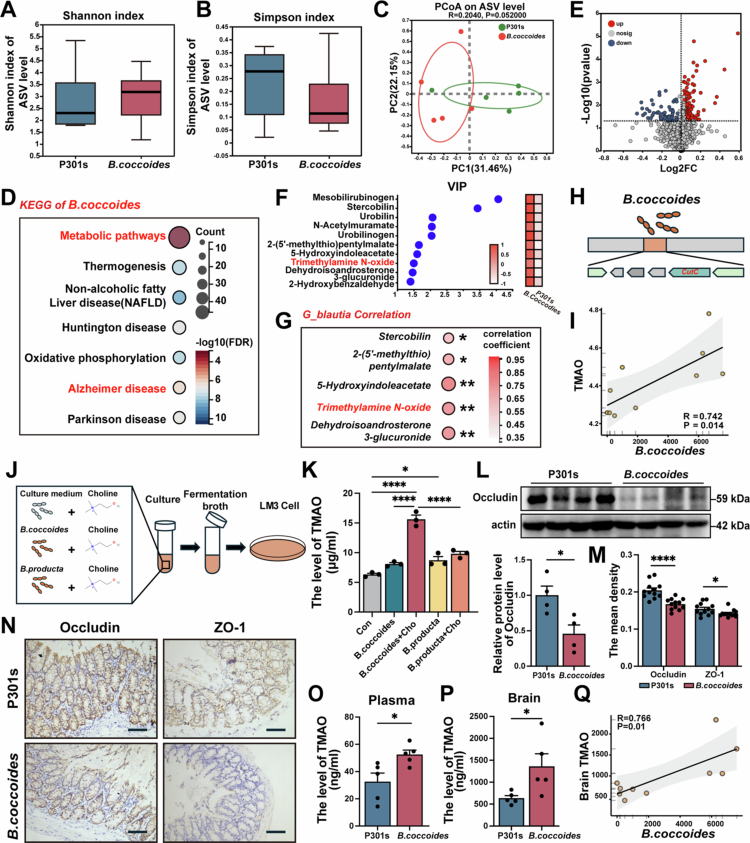
*B. coccoides* supplementation promoted the production of TMAO in both AD mouse models and *in vitro*. (A) and (B) α-diversity measurement of the Shannon index and Simpson index between P301s and *B. coccoides* group (*n* = 5). (C) PCoA showed variations in the gut microbiota composition between the two groups (*n* = 5). (D) KEGG pathway enrichment analysis of *B. coccoides* functional genes. (E) Volcano plot displayed of differential metabolites between the two groups (*n* = 5), FDR < 0.05. (F) VIP analysis revealed that TMAO was an important differential metabolite (*n* = 5). (G) Bubble chart displayed the significant Spearman correlation between *Blautia* abundance and differential metabolites from the VIP analysis. The gradient colors and the size of the circle represent the correlation coefficient. (H) Functional sequence of C*utC* indicated the bacterial biology of *B. coccoides*. (I) Linear regression analysis of *B. coccoides* and the fecal TMAO levels. (J) Illustration of the experimental setup in which the fermentation broth from a 24-h co-culture of *B. coccoides* or *B. producta* and 50 mg/L Choline was applied to LM3 cells. (K) Quantitative analysis of TMAO level among Con, *B. coccoides, B. coccoides* + Choline, *B. producta and B. producta* + Choline groups (*n* = 3). (L) Representative Western blot images and quantitative analysis of Occludin in P301s and *B. coccoides* group (*n* = 4). (M) Quantitative analysis of Occludin and ZO-1 levels in the P301s group and *B. coccoides* group (*n* = 4). (*N*) Representative immunohistochemical images of Occludin and ZO-1 in the P301s group and *B. coccoides* group. The image above: magnification 200×. Scale bar = 100 μm. (O) Quantitative analysis of TMAO level in plasma between two groups (*n* = 5). (P) Quantitative analysis of TMAO levels in the brain between the two groups (*n* = 5). (Q) Linear regression analysis of *B. coccoides* and brain's TMAO levels. The error bars indicate the SEM, *p < 0.05 and ****p < 0.0001.

The results of 16 s rRNA analysis, there were no significant differences in α-diversity indices, including the Simpson index and Shannon index, between the *B. coccoides*-treated group and P301s group (Figure 2A and B). Moreover, the results of the ASV level PcoA results showed no significant difference between the groups (*p* > 0.05, Figure 2C). These findings indicated that *B. coccoides* treatment did not alter the structure of the gut microbiota in P301s mice.

Metabolites produced or induced by gut bacteria might signal outside the gastrointestinal tract and regulate brain functions through the gut‒brain axis.^32^ Subsequently, we focused on vital metabolites produced by *B. producta* that mediated the pathogenesis of AD. To identify the key metabolite, we performed functional gene analysis of *B. coccoides* and LC‒MS analysis in *vivo* and *in vitro*. KEGG pathway enrichment analysis of *B. coccoides* functional genes, using public sequencing data (https://www.atcc.org/) was performed. The results showed enrichment in metabolic and Alzheimer's disease pathways in *B. coccoides* functional genes (FDR < 0.05, Figure 2D). We further investigated fecal gut microbiota metabolites by untargeted metabolomic analysis. The results of Volcano plot showed the distribution of differential metabolites between the two groups (Figure 2E). According to the results of KEGG pathway enrichment analysis of the differential metabolite showed pathways, including energy metabolism, were significantly enriched (FDR < 0.05, Figure S1A). Furthermore, KEGG pathway enrichment analysis of the 158 upregulated differential metabolites demonstrated significant enrichment in several pathways, including Nervous system, energy metabolism and neurodegenerative disease were significantly enriched (FDR < 0.05, Figure S1B). VIP analysis of differential metabolites within metabolic pathways to identify specific differential metabolites, such as TMAO (VIP > 1, fold change > 1 and *p*-value < 0.05, Figure 2F). Correlation analysis revealed a significant positive correlation between 5 VIP differential metabolites, including TMAO, and the abundance of *g_Blautia* (*p* < 0.05, Figure 2G). Analysis of functional gene fragments revealed that *B. coccoides* harbors CutC, a key enzyme involved in TMA metabolism (Figure 2H). Furthermore, linear regression analysis showed that *B. coccoides* abundance was significantly positively correlated with the fecal TMAO level, indicating that TMAO may be the key metabolites of *B. coccoides* (*p* < 0.05, Figure 2I). To further confirm that *B. coccoides* could produce TMA/TMAO, *B. coccoides* was co-cultured with Choline *in vitro*, and the resulting fermentation broth was used to treat HCC-LM3 cells, which express the FMO3 enzyme capable of converting TMA to TMAO (Figure 2J and S1D–K). The results showed that the TMA level in fermentation broth was significantly increased in *B. coccoides* + Choline group (*p* < 0.05, Figure S1H), and the TMAO level in the LM3 medium was significantly higher in the *B. coccoides* + Choline group compared to the Con group (*p* < 0.05, Figure 2K). *B. coccoides* + Choline group had a higher TMAO level compared to *other species of B. productia* + Choline group (*p* < 0.05, Figure 2K). These findings suggested that *B. coccoides* could directly produce TMAO.

Additionally, we investigated whether *B. coccoides* impacts gut barrier damage, the levels of gut barrier tight junction (TJ) proteins, including Occludin and ZO-1, were assessed. The results showed that *B. coccoides* treatment significantly decreased the levels of Occludin and ZO-1 in P301s mice (*p* < 0.05, Figure 2L–N), indicated *B. coccoides* could exacerbate gut barrier damage and increase in intestinal permeability of AD. These results indicated that *B. coccoides* produced TMAO, intestinal permeability allowing TMAO into systemic circulation.

Furthermore, we hypothesized that the abundance of *B. coccoide*s might correlate with serum and brain TMAO levels. We conducted LC‒MS analysis to determine TMAO level in serum and brain samples obtained from *B. coccoides*-treated mice and P301s mice. We observed significantly higher TMAO levels of serum and brain in *B. coccoides*-treated mice than in P301s mice (*p* < 0.05, Figure 2O and P). Further, linear regression showed a significantly positive correlation between *B. coccoides* abundance and cerebral TMAO level in P301s mice (*p* < 0.05, Figure 2Q).

### Elevated serum TMAO levels were correlated with dementia severity in AD patients and TMAO supplementation could promote oxidative stress *in vitro*

**Figure 3.**
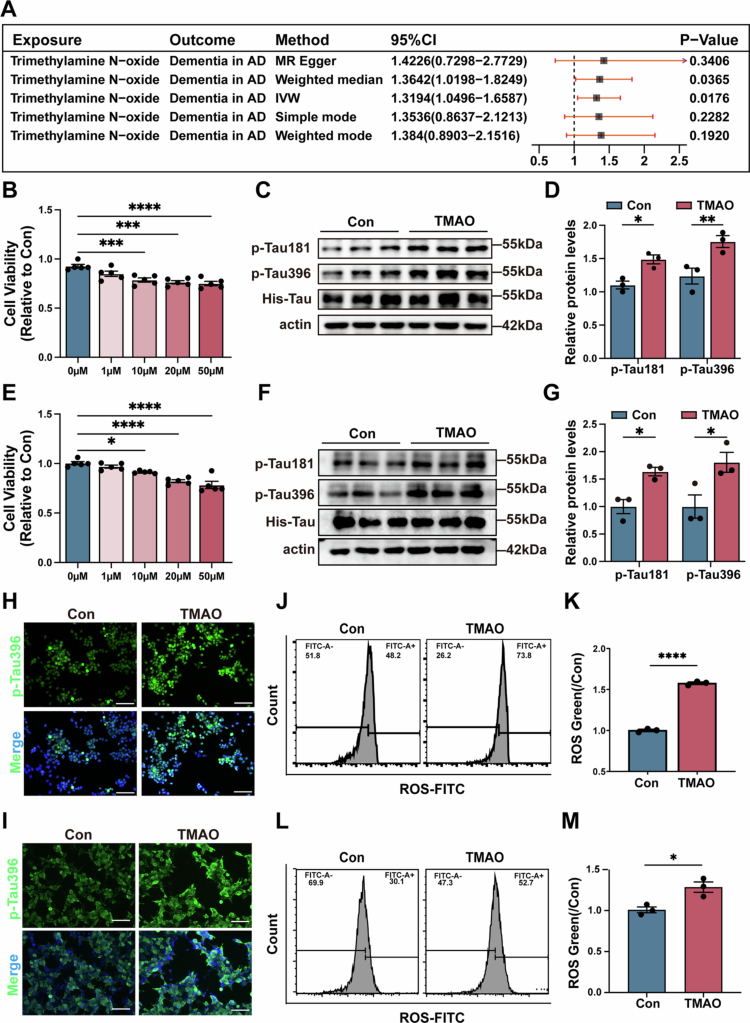
The TMAO level was correlated with dementia severity in AD patients, and TMAO treatment promoted Tau phosphorylation and oxidative stress *in vitro*. (A) Forest plot of MR analyses for the associations between TMAO and Dementia in AD, points represent beta estimates and 95% CI. (B) P301L-N2a cells were treated with 0, 1, 10, 20 and 50 μM TMAO, and cell viability was assessed using the CCK-8 method (*n* = 5). (C) Representative Western blot images of p-Tau181 and p-Tau396 of Con group and TMAO group in P301L-N2a cells. (D) Quantitative analysis of p-Tau181 and p-Tau396 levels (*n* = 3). The Con group was used as a reference value. (E) P301L-293T cells were treated with 0, 1, 10, 20 and 50 μM TMAO, and cell viability was assessed using the CCK-8 method (*n* = 5). (F) Representative Western blot images of p-Tau181 and p-Tau396 of Con group and TMAO group in P301L-293T cells. (G) Quantitative analysis of p-Tau181 and p-Tau396 levels (*n* = 3). The Con group was used as a reference value. (H) Representative immunofluorescence images of p-Tau396 of Con group and TMAO group in P301L-N2a cells. (I) Representative immunofluorescence images of p-Tau396 of Con group and TMAO group in P301L-293T cells. (J) Flow cytometry of ROS levels in Con and TMAO treated group of P301L-N2a. (K) Quantitative analysis of ROS in the two groups of P301L-N2a (*n* = 3). (L) Flow cytometry of the ROS levels in the Con and TMAO treated group of P301L-293T. (M) Quantitative analysis of ROS in the two groups of P301L-293T (*n* = 3). The image above: magnification 200×. Scale bar = 100 μm. The error bars indicate the SEM, *p < 0.05, **p < 0.01, ***p < 0.01, and ****p < 0.0001.

Elevated TMAO levels are associated with several human diseases, including AD.^33^,^34^ We assessed the effect of TMAO on dementia severity in AD patients. Two-sample MR analysis revealed a positive causal relationship between plasma TMAO levels and dementia severity in AD patients (*p* < 0.05, Figures 3A and S2A–D). Collectively, these clinical data demonstrated an increase in TMAO levels in AD patients, which also suggested a potential role for TMAO in the pathogenesis of AD.

*In vitro* experiments, we used Neuro-2a (P301L-N2a) and HEK-293T (P301L-293T) cells transfected with MAPT P301L as model cells. Neuro-2a (Figure 3B) and HEK-293T (Figure 3E) cells were treated with 0, 1, 10, 20 and 50 μM TMAO, and cell viability was assessed by CCK-8 (*p* < 0.05). We observed that the effect was most significant at a concentration of 20 μM, which was well below the IC₅₀ threshold (Figure S3B and C) while already exerting biological effects (Figure S3D–G), and 20 μM TMAO was used in subsequent cell experiments. We further confirmed the effect of TMAO on promoting Tau phosphorylation *in vitro*, and the levels of Tau phosphorylation at the T181 and S396 sites were assessed by Western blot. The results showed that TMAO treatment markedly increased the levels of p-Tau181 and p-Tau396 in P301L-N2a (*p* < 0.05, Figure 3C and D) and P301L-293T cells (*p* < 0.05, Figure 3F and G). Furthermore, immunofluorescence analysis showed TMAO treatment increased the level of Tau phosphorylation p-Tau181 and p-Tau396 in P301L-N2a (Figures 3H and S3H) and P301L-293T cells (Figures 3I and S3I). These findings indicated that TMAO could promote Tau phosphorylation in AD.

TMAO has been implicated in promoting oxidative stress,^35^,^36^ which further exacerbates the Tau phosphorylation driven by ROS and lipid peroxidation. We investigated whether TMAO promoted oxidative stress, and the ROS level was assessed by Flow cytometry. The results showed that TMAO treatment significantly increased the level of ROS in P301L-N2a cells (*p* < 0.05, Figure 3J and K) and P301L-293 T cells (*p* < 0.05, Figure 3L and M). These results suggest that TMAO promoted ROS production, which contribute to oxidative stress in AD.

### Analysis of key signaling pathway regulated by TMAO in AD

**Figure 4.**
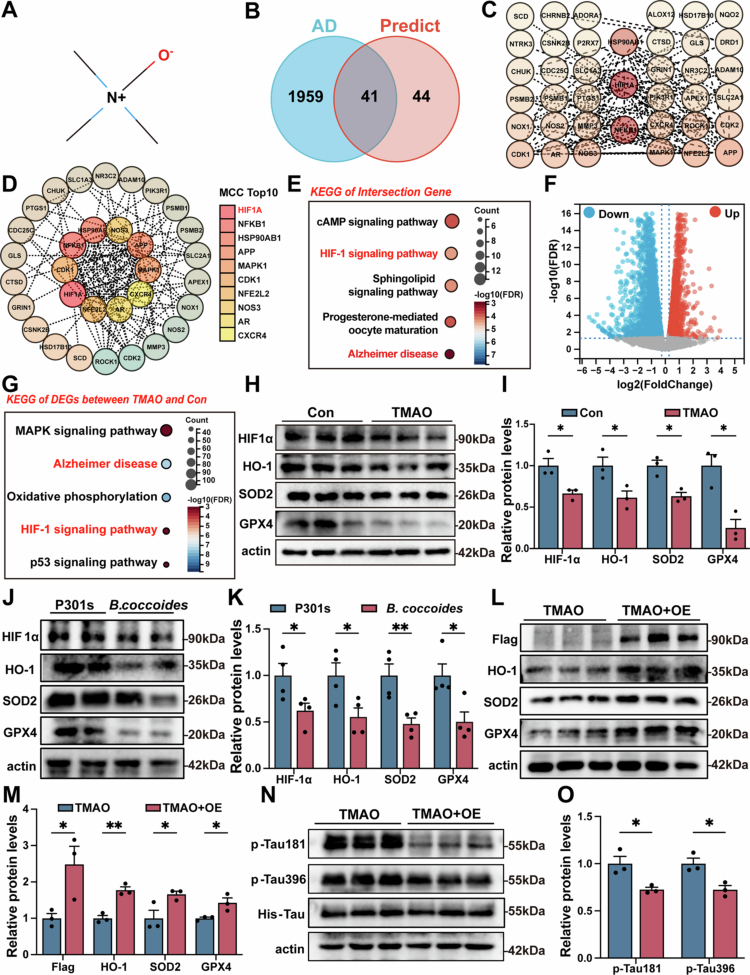
TMAO promoted oxidative stress by suppressing the HIF1α signaling pathway. (A) Chemical structure of TMAO. (B) Venn diagram showed overlap between TMAO binding targets and AD-related genes. (C) PPI network diagram of TMAO and AD intersection genes (interaction score > 0.7). (D) MCC screening of overlap targets identified the top 10 core targets with the shortest interaction paths in the PPI network. (E) KEGG pathway enrichment showing that the HIF1 and Alzheimer disease pathways were significantly enriched in overlap genes, FDR < 0.05 was considered statistically significant. (F) Volcano plot showing the distribution of gene expression level differences between the Con and TMAO group. FDR < 0.05 were screened as differentially expressed genes. (G) KEGG pathway enrichment showing that the HIF1 and Alzheimer's disease pathways were significantly enriched in DEGs between the Con and TMAO groups; FDR < 0.05 was considered statistically significant. (H) Representative Western blot analysis of HIF1 pathway in the Con and TMAO group, including HIF1α, HO-1, SOD2 and GPX4. (I) Quantification of HIF pathway between two groups (*n* = 3); the Con group was used as a reference value. (J) Representative Western blot of HIF1 pathway in P301s and *B. coccoides* group, including HIF1α, HO-1, SOD2 and GPX4. (K) Quantification of HIF pathway between P301s and *B. coccoides* group (*n* = 4); the P301s group was used as a reference value. (L) Representative Western blot of HIF1 pathway in the TMAO and TMAO + OE group, including HIF1α, HO-1, SOD2 and GPX4. (M) Quantification of HIF pathway between TMAO and TMAO + OE groups (*n* = 3); the TMAO group was used as a reference. (N) Representative Western blot images of AT8, p-Tau181 and p-Tau396 in the two groups. (O) Quantitative analysis of AT8, p-Tau181 and p-Tau396 levels between TMAO group and TMAO + OE group (*n* = 3); TMAO group was used as a reference. The error bars indicate the SEM, *p < 0.05 and **p < 0.01.

To elucidate the mechanisms underlying the promotion of oxidative stress after TMAO treatment, we performed drug target analysis and RNA-seq to identify key signal pathway related to oxidative stress. The chemical structure of TMAO was showed in Figure 4A. The SMILES data for TMAO were obtained from PubChem and analyzed with SuperPred (https://prediction.charite.de/) to identify potential target interactions. GeneCards (https://www.genecards.org/) was used to find genes linked to AD. The Venn diagram shows that there are 41 overlapping genes between AD-associated genes and predicted TMAO targets (Figure 4B). To identify the core targets of TMAO exacerbated AD, we performed a PPI network analysis based on the STRING database (Figure 4C) and CytoHubba MCC algorithm of Cytoscape. Finally, the top 10 hub genes, including HIF1α, were identified (Figure 4D). Moreover, KEGG enrichment analysis of the TMAO targets in Figure 4B showed HIF1 pathway and Alzheimer's disease pathway were significantly enriched (FDR < 0.05, Figure 4E). In addition, RNA-seq analysis was performed to identify differentially expressed genes (DEGs) in Neuro-2a cells with or without TMAO treatment (|log2FC| > 1.2, *p* < 0.05). Volcano plot showed the distribution of DEGs between the two groups (Figure 4F). Then, KEGG analysis showed HIF1 signaling pathway and Alzheimer's disease pathway were also significantly enriched in the DEGs of the two groups (FDR < 0.05, Figure 4G), indicating that the HIF1 pathway may play an important role in TMAO exacerbated AD. Therefore, after 18 h of TMAO treatment followed by 6 h of hypoxic exposure, Western blot analysis was performed to assess the changes in the HIF1α signaling pathway in P301L-N2a cells. The result showed that the protein expression of HIF1α, HO-1, SOD2 and GPX4 were significant decreased in the TMAO-treated group (*p* < 0.05, Figure 4H and I). Additionally, the protein expression levels of HIF1α, HO-1, SOD2, and GPX4 were also significantly decreased in P301s mice treated with *B. coccoides* (*p* < 0.05, Figure 4J and K).

To further confirm the role of TMAO in promoting Tau phosphorylation via the HIF1α-mediated HIF1 signaling pathway, P301L-293T cells were overexpressed with HIF1α and treated with TMAO. After hypoxic exposure, the levels of HIF1α, HO-1, SOD2 and GPX4 were significantly rescued in the TMAO + OE (overexpression) group (*p* < 0.05, Figure 4L and M). Additionally, the protein levels of Tau phosphorylation, including p-Tau181 and p-Tau396, were reduced in the TMAO + OE group compared to the TMAO group (*p* < 0.05, Figure 4N and O).

To further confirm the role of TMAO in promoting Tau phosphorylation in vivo, P301s mice were treated with TMAO for 21 d. Our results showed that the intervention with TMAO promoted significant deterioration of cognitive function in P301s mice. In the ORT, the mice in the P301L + TMAO group showed a significant decrease in the discrimination index compared with the P301L group (*p* < 0.01, Figure S5A). In the Barnes maze, the P301L + TMAO mice exhibited a longer escape latency (*p* < 0.05, Figure S5B) and less time spent in the target quadrant (*p* < 0.01, Figure S5C). Immunohistochemical analysis demonstrated that TMAO treatment significantly increased TUNEL^+^ positive cells in the cortex (*p* < 0.01, Figure S5D and E) and Tau phosphorylation in P301L mice (*p* < 0.05, Figure S5F and G). Consistently, the Western blot results revealed elevated levels of p-Tau at both Thr181 and Ser396 in the P301L + TMAO group (*p* < 0.05, Figure S5H–J). Additionally, TMAO treatment rescued the expression of HIF-1α, HO-1, SOD2 and GPX4 (*p* < 0.05, Figure S5K and L), suggesting enhanced oxidative stress and altered redox homeostasis.

### TMAO promoted Tau phosphorylation via HIF1α interaction

**Figure 5.**
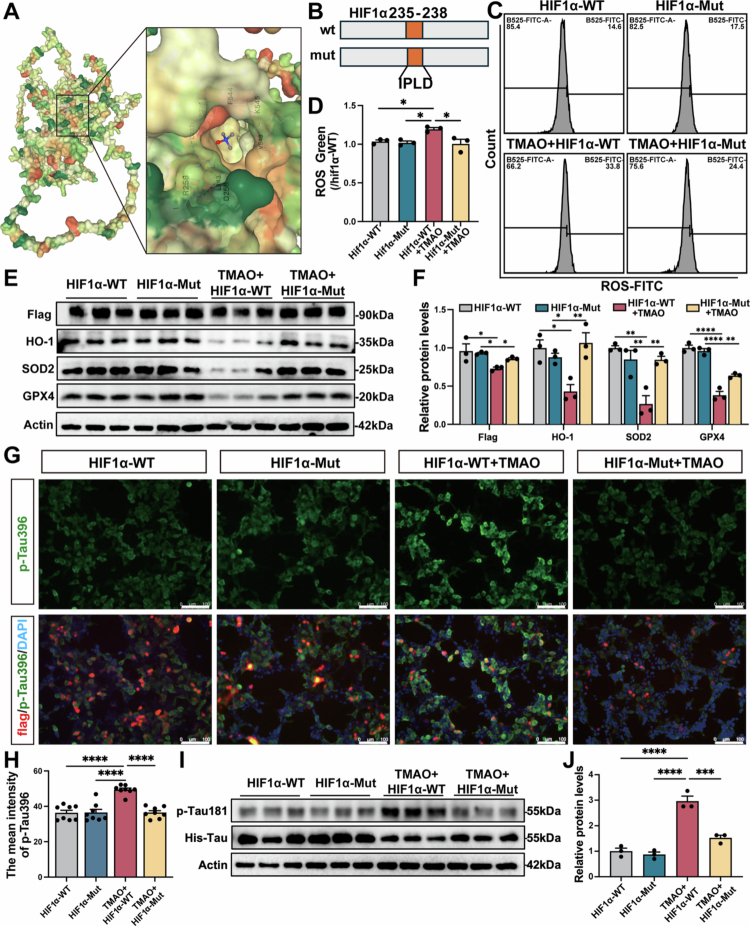
TMAO promoted Tau phosphorylation via the HIF1α interaction. (A) AutoDock simulation of the TMAO-HIF1α interaction. (B) Diagram of the HIF1α deletion mutant at hydrogen bond binding sites 235–237 with TMAO. (C) Flow cytometry of the ROS level of P301L cells in HIF1α-WT, HIF1α-mut, TMAO + HIF1α-WT and TMAO + HIF1α-mut group. (D) Quantitative analysis of ROS among the four groups (*n* = 3). (E) Representative Western blot of the HIF1 pathway in the four groups. (F) Quantification of the HIF pathway; the HIF1α-WT group was used as a reference value (*n* = 3). (G) Representative immunofluorescence images of p-Tau396 in the four groups. The image above: magnification 200×. Scale bar = 100 μm. (H) Quantification of p-Tau396 levels (*n* = 3). (I) Representative Western blot images of p-Tau181 in the four groups. (J) Quantitative analysis of p-Tau181 levels; the HIF1α-WT group was used as a reference value (*n* = 3). Error bars indicate the SEM, *p < 0.05, **p < 0.01, ***p < 0.001, and ****p < 0.0001.

To explore how TMAO affects the HIF1 pathway, we examined its interaction with the MCC core target HIF1α. The binding results revealed that TMAO exhibited binding activity with HIF1α at 235–238 sites (Figure 5A). Therefore, we constructed wild-type HIF1α (HIF1α-WT) and 235–238 deleted mutant HIF1α (HIF1α-Mut) overexpression plasmids to clarify whether TMAO regulates AD through the HIF1α interaction (Figure 5B). HIF1α-WT and HIF1α-Mut P301L-293T cells were treated with or without TMAO. Then, Flow cytometry showed a significant increase in ROS level in the TMAO + HIF1α-WT group compared to HIF1α-WT. However, ROS levels were rescued in the TMAO + HIF1α-Mut group compared to TMAO + HIF1α-WT (*p* < 0.05, Figure 5C and D). Then, Western blot analysis showed a significant reduction in the protein levels of HIF1α, HO-1, SOD2, and GPX4 in the TMAO + HIF1α-WT group compared to the HIF1α-WT group but not in the TMAO + HIF1α-Mut group (*p* < 0.05, Figure 5E and F), and the protein levels of Tau phosphorylation, including p-Tau181 and p-Tau396, were decreased in the TMAO + HIF1α-Mut group compared with TMAO + HIF1α-WT group (*p* < 0.05, Figure 5G–J). Overall, these data confirmed an interaction between TMAO and HIF1α, suggesting that TMAO mechanistically contributes to oxidative stress in AD through the inhibition of the HIF1α signal pathway.

## Discussion

The gut microbiota plays a pivotal role in host health, which was linked to brain diseases.^37^,^38^ Here, we observed a significant increase of *B. coccoides* abundance in AD patients and demonstrated that supplementing with *B. coccoides* could exacerbate cognitive impairment and Tau phosphorylation in AD model animals. In fact, with an increasing understanding of the complex interactions between *B. coccoides* and its host, the exploration on its metabolite is urgently required to confirm the exact role of *B. coccoides* in AD pathogenesis. Notably, we identified TMAO as a key metabolite of *B. coccoides in vivo* and *in vitro*, and demonstrated that TMAO could exacerbate Tau phosphorylation, indicating that *B. coccoides* might be an important contributing factor in the pathogenesis of AD. Mechanistically, TMAO acts through binding HIF1α, inhibiting the HIF1α signal pathway, thereby promoting oxidative stress and aggravating AD pathologies. This study provided a novel target for therapeutic interventions of AD.

In this study, we observed that the abundance of *B. coccoides* in AD patients was significantly higher than that in HC, which was positively correlated with the serum p-Tau level. *B. coccoides*, a species of the *Blautia* genus, is a facultative anaerobe that resides in the human gastrointestinal tract,^19^ which was closely associated with certain diseases, such as type I diabetes ^39^ and irritable bowel syndrome.^40^ In this study, we demonstrated that *B. coccoides* supplement could effectively exacerbate cognitive impairment and Tau phosphorylation in the P301S mice, indicating that *B. coccoides* might be involved in the pathogenesis of AD. Accumulating evidence has revealed that metabolites produced or induced by gut bacteria, such as short-chain fatty acids, bile acid and TMAO, might be signals emitted from outside the gastrointestinal tract,^41^,^42^ which contributed to mechanistically understand the microbiota-host crosstalk. To explore the underlying mechanisms of *B. coccoides* in aggravating AD pathologies, we investigated the metabolites altered by *B. coccoides*. Notably, we discovered that *B. coccoides* directly produced TMAO, and found TMAO-producing metabolic enzyme genes in the genome of *B. coccoides* and demonstrated that *B. coccoides* abundance was significantly positively correlated with fecal TMAO levels. More importantly, we found that the TMAO levels of the serum and brain in *B. coccoides*-treated mice was significantly increased, and there was a positive correlation between *B. coccoides* abundance and TMAO level in the brain, indicating that *B. coccoides* could directly produce TMAO that mediated the harmful effects.

In this study, we focused on the microbial metabolite TMAO to clarify its mechanism on AD pathologies. We observed an increase in the plasma TMAO level of AD patients, which was positively correlated with dementia severity. In addition, previous work have reported an increase of cerebrospinal fluid TMAO levels in AD patients and mild cognitive impairment (MCI) subjects.^35^ Moreover, we found that supplementation with TMAO could increase Tau phosphorylation and promote oxidative stress *in vitro*. TMAO has also been intimately linked to oxidative stress.^35^,^36^ Oxidative stress has been recognized as a hallmark of AD progression.^43^ The excessive production of reactive oxygen species (ROS) aggravates oxidative stress, which act to further enhance hyperphosphorylation of tau protein.^44^,^45^ We further confirm that TMAO acts through HIF1α, which directly binds to HIF1α at 235−238 sites, inhibiting HIF1α signal, ultimately contributing to AD pathology. HIF1α, a highly conserved heterodimeric transcription factor, plays a pivotal role in regulating oxygen homeostasis.^46^ Patients with AD have been shown to have reduced levels of HIF1α, and decreased HIF1α levels are associated with increased tau protein phosphorylation.^47^ It was reported that TMAO inhibited cardiac angiogenesis by reducing HIF1α protein levels, ultimately aggravating cardiac dysfunction.^48^ These findings suggested that *B. coccoides-*derived metabolite TMAO could directly bind to HIF1α, ultimately contributing to Tau phosphorylation, indicating that *B. coccoides* could directly produce TMAO that mediated the harmful effects via inhibiting HIF1α signal pathway. However, exploring the effects and mechanisms of several strains of *B. coccoides* will be more comprehensive and conclusive. A further study to explore the effects of different *B. coccoides* strains via a germ-free animal model is necessary.

In conclusion, we demonstrated that *B. coccoides*-derived TMAO promotes oxidative stress, ultimately promoting Tau phosphorylation in AD. These actions are associated with the binding of TMAO to HIF1α to inhibit HIF1α signal pathway. Furthermore, this study highlighted TMAO-mediated HIF1α signaling cascade mediates the crosstalk between *B. coccoides* and the host, exacerbating AD progression. This study provided new insights for microbe/metabolite-based intervention strategies for addressing AD.

## Supplementary Material

Supplementary material Table S1

Supplementary material Supplement Figure

## Data Availability

The data that support the findings of this study are available from the corresponding author upon reasonable request. The datasets analyzed in the present study can be downloaded from https://prediction.charite.de/, https://www.finngen.fi/en, https://www.ebi.ac.uk/gwas/downloads/summary-statistics.
