# Supplementary material for: Blautia coccoides-derived metabolite trimethylamine-N-oxide exacerbates Alzheimer's disease progression via targeting HIF1α signaling
Source: Gut Microbes. 2025 Dec 29;18(1):2605768. doi: 10.1080/19490976.2025.2605768 (PMC12758303; doi:10.1080/19490976.2025.2605768)
Supplement: Supplementary material — Table S1 [file KGMI_A_2605768_SM2939.docx]

**Table S1.** **Primer sequences**

| **Primer Name** |  | **Sequence (5' to 3')** |
| --- | --- | --- |
| *Blautia.coccoides* | Forward | ACATGCATGATCCGACAGAA |
|  | Reverse | ACCGTCTAAAAACGGGGTTC |
| V3-V4 | Forward | ACTCCTACGGGAGGCAGCA |
|  | Reverse | GGACTACHVGGGTWTCTAAT |
