# Supplementary material for: Blautia coccoides-derived metabolite trimethylamine-N-oxide exacerbates Alzheimer's disease progression via targeting HIF1α signaling
Source: Gut Microbes. 2025 Dec 29;18(1):2605768. doi: 10.1080/19490976.2025.2605768 (PMC12758303; doi:10.1080/19490976.2025.2605768)
Supplement: Supplementary material — Supplement Figure [file KGMI_A_2605768_SM2940.docx]

**
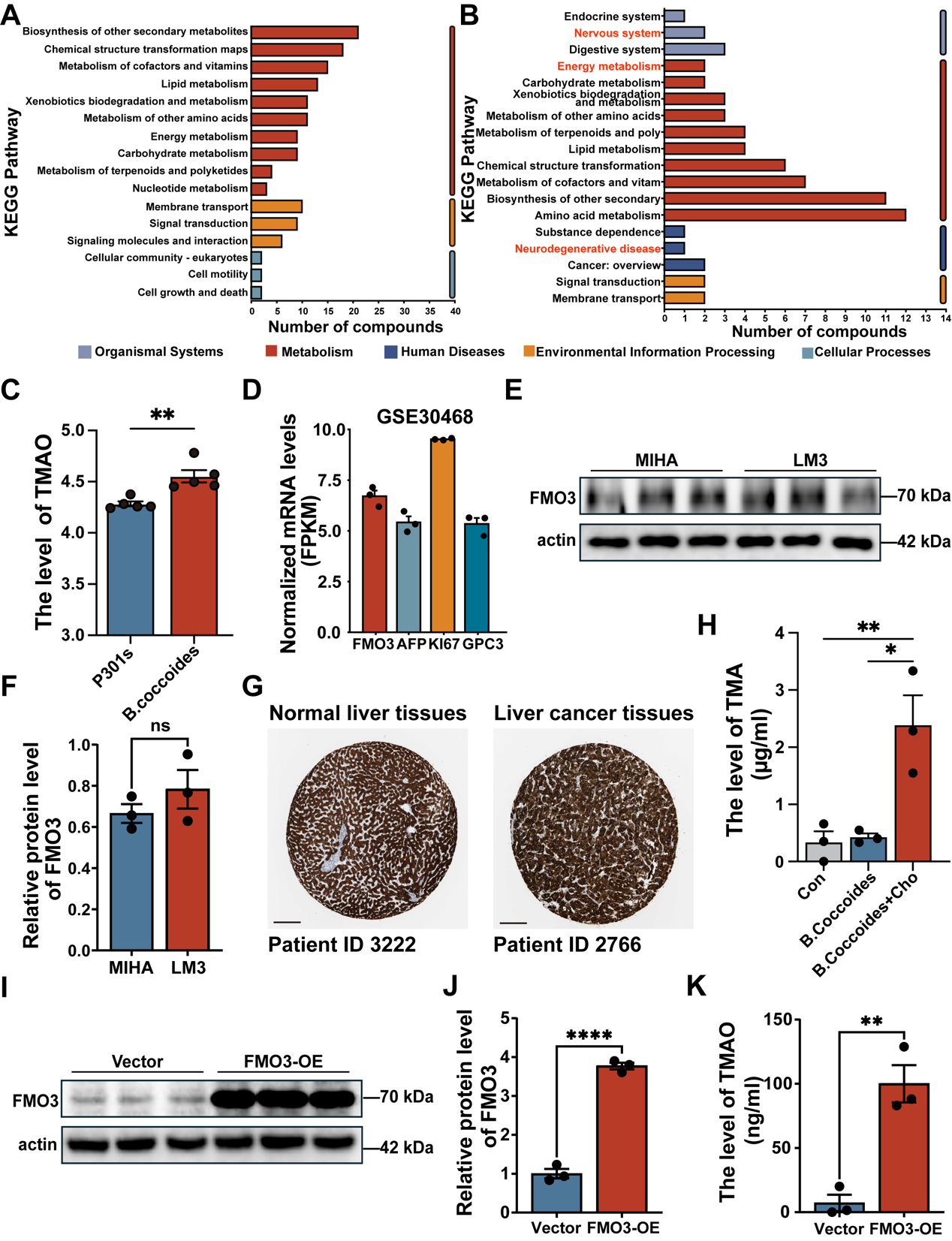
Figure S1 Analysis of differential metabolites in P301s and *B. coccoides* groups, and evaluation of the TMA-to-TMAO conversion capacity of FMO3 *in vitro*.** (A) KEGG enrichment analysis of differential metabolite showed metabolic pathways, including energy metabolism, were significantly enriched. FDR< 0.05 was considered statistically significant. (B) KEGG pathway enrichment analysis of the 158 upregulated differential metabolites demonstrated significant enrichment in several metabolic pathways, including Nervous system, energy metabolism and neurodegenerative disease were significantly enriched. FDR< 0.05 was considered statistically significant. (C) Quantitative analysis of TMAO level in feces between P301s group and *B. coccoides* group (n = 5). (D) Expression of FMO3 in HCC-LM3 cells, using AFP, KI67, and GPC3 as reference markers. (E) Representative Western blot images of FMO3 between normal human liver cells (MIHA cells) group and HCC-LM3 group. (F) Quantitative analysis of FMO3 level between MIHA cells group and HCC-LM3 group (n = 3). (G) Representative Immunohistochemistry images of FMO3 staining in Normal liver tissue and Liver cancer tissue from The Human Protein Atlas (https://www.proteinatlas.org/). Scar bar = 200 µm. (H) Quantitative analysis of TMA level among L-carnitine, B. coccoides, B. coccoides groups+ L-carnitine groups, where the fermentation broth from a 24-hour co-culture of B. coccoides and 50 mg/L L-carnitine (n = 3). (I) Representative Western blot images of FMO3 between Vector group and FMO3-OE group. (J) Quantitative analysis of FMO3 level between Vector group and FMO3-OE group (n = 3). (K) Quantitative analysis of TMAO level between Vector group and FMO3-OE group, which were treated with TMA (n = 3). Error bars indicate the SEM, * *P* < 0.05, ** *P* <0.01.


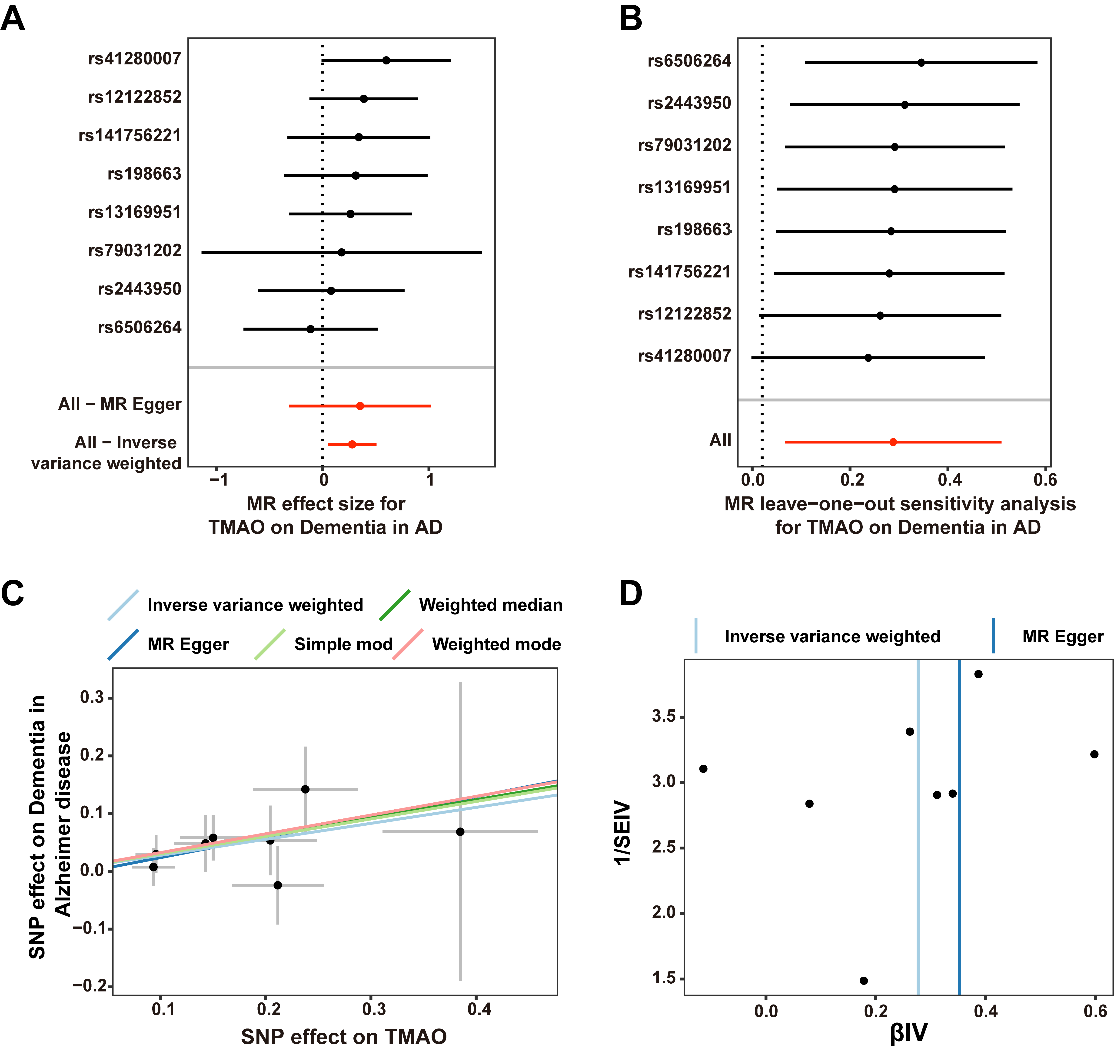


**Figure S2 MR analyses for the associations between TMAO and Dementia in AD.** (A) Forest plot of the causal effect of TMAO on dementia in AD. (B) Leave-one-out analysis of the causal effect of TMAO on Dementia in AD. (C) Scatterplots for MR analyses of the causal effect of TMAO on dementia in AD. The slope of each line corresponds to the estimated MR effect per method. (D) Funnel Plot of Heterogeneity in MR Analysis.


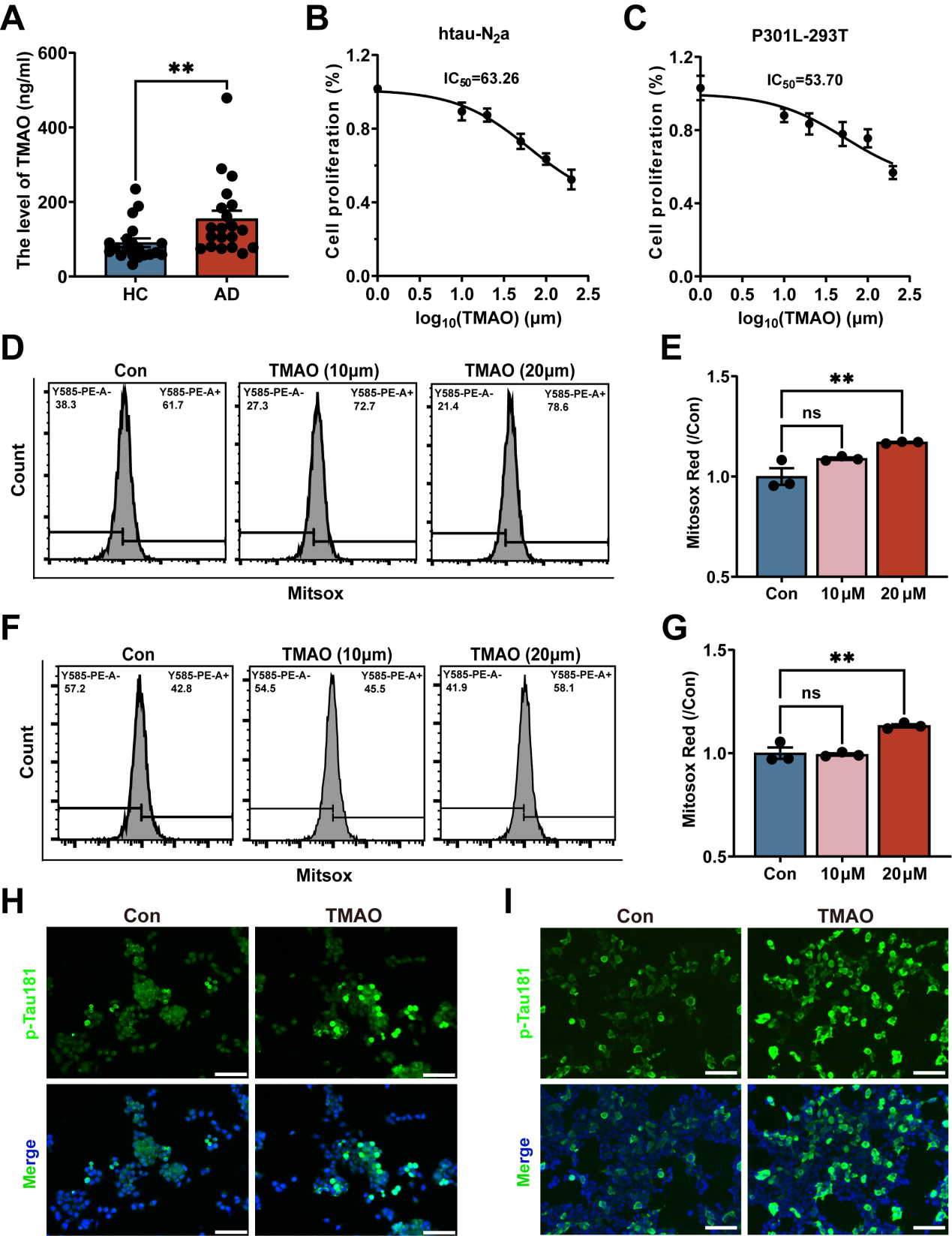


**Figure S3 Circulating TMAO level was increased in AD patients and TMAO treatment promoted Tau phosphorylation and oxidative stress *in vitro*.** (A) Quantitative analysis of Circulating TMAO level in plasma between HC groups and AD patients (n = 20). (B) Dose–response experiment of P301L-N2a cells treated with TMAO at concentrations ranging from 0 to 200 µM, and calculation of the corresponding IC₅₀ value (n = 3). (C) Dose–response experiment of P301L-293T cells treated with TMAO at concentrations ranging from 0 to 200 µM, and calculation of the corresponding IC₅₀ value (n = 3). (D - E) Flow cytometry of MitoSOX levels in Con and 10μM TMAO treated group and 20 μM TMAO treated group of P301L-N2a cells (n = 3). (F - G) Flow cytometry of MitoSOX levels in Con and 10 μM TMAO treated group and 20 μM TMAO treated group of P301L-293T cells (n = 3). (H) Representative immunofluorescence images of p-Tau 181 of Con group and TMAO group in P301L-N2a cells. The image above: Magnification 200 ×. Scale bar = 100 μm. (I) Representative immunofluorescence images of p-Tau 181 of Con group and TMAO group in P301L-293T cells. The image above: Magnification 200 ×. Scale bar = 100 μm. Error bars indicate the SEM, * *P* < 0.05, ** *P* <0.01.

**
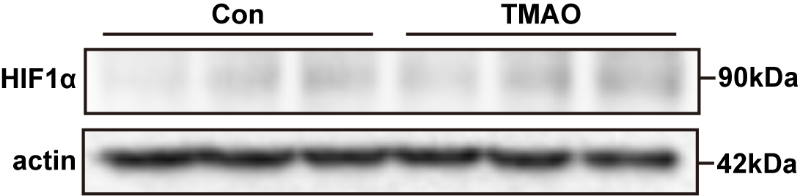
**

**Figure S4 HIF-1α expression remained low under normoxic conditions.** Representative Western blot images of HIF1α between Con and TMAO group under normal oxygen conditions (n = 3).

*
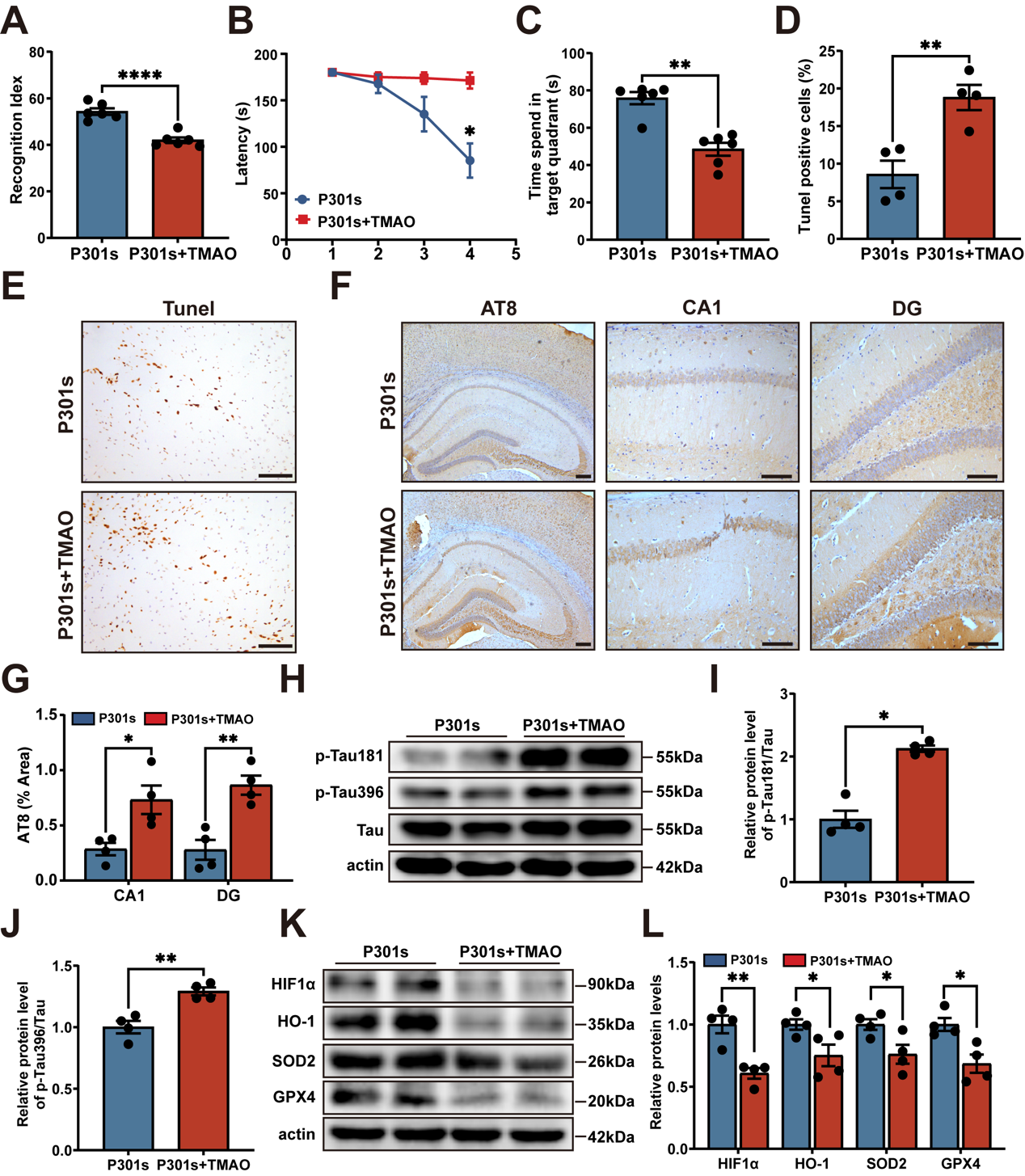
*

**Figure S5 TMAO supplementation aggravated cognitive function and pathological changes in P301s mice.** (A) Comparisons of Discriminant index between P301s and P301s + TMAO group (n = 6). (B) Comparisons of Latency in Barnes maze test between P301s and P301s + TMAO group (n = 6). (C) Comparisons of time spend in target quadrant in Barnes maze test between two groups (n = 6). (D) Quantitative analysis of Tunel^+^ cells in (E, n = 4). (E) Representative Immunohistochemistry images of Tunel staining in two groups. The image above: Magnification 200 × and Scale bar = 100 μm. (F) Representative immunohistochemical images of AT8 in two groups. The image above: Magnification 50× and 200 ×. 50× Scale bar = 200 μm, and 200 × Scale bar = 100 μm. (G) Quantitative analysis of AT8 level between two groups (n = 4). (H) Representative Western blot images of p-Tau181 and p-Tau396 in in two groups. (I) R Quantitative analysis of p-Tau181 and p-Tau396 levels between two groups (n = 4). (J) Representative Western blot of HIF1 pathway in two groups, including HIF1α, HO-1, SOD2 and GPX4. (K) Quantification of HIF pathway between two groups; P301s group was used as a reference value (n = 4). Error bars indicate the SEM, * *P* < 0.05, ** *P* < 0.01, *** *P* < 0.001.
